# Differences in cortical processing of facial emotions in broader autism phenotype

**DOI:** 10.1371/journal.pone.0262004

**Published:** 2022-01-18

**Authors:** Patricia Soto-Icaza, Brice Beffara-Bret, Lorena Vargas, Francisco Aboitiz, Pablo Billeke

**Affiliations:** 1 Laboratorio de Neurociencia Social y Neuromodulación, Centro de Investigación en Complejidad Social (neuroCICS), Facultad de Gobierno, Universidad del Desarrollo, Santiago, Chile; 2 LPPL–EA 4638, Université de Nantes, Nantes, France; 3 Centro del Niño, Clínica Alemana, Santiago, Chile; 4 Laboratorio de Neurociencias Cognitivas, Departamento de Psiquiatría, Centro Interdisciplinario de Neurociencias, Facultad de Medicina, Pontificia Universidad Católica de Chile, Santiago, Chile; Universita degli Studi di Udine, ITALY

## Abstract

Autism Spectrum Disorder (ASD) is a heterogeneous condition that affects face perception. Evidence shows that there are differences in face perception associated with the processing of low spatial frequency (LSF) and high spatial frequency (HSF) of visual stimuli between non-symptomatic relatives of individuals with autism (broader autism phenotype, BAP) and typically developing individuals. However, the neural mechanisms involved in these differences are not fully understood. Here we tested whether face-sensitive event related potentials could serve as neuronal markers of differential spatial frequency processing, and whether these potentials could differentiate non-symptomatic parents of children with autism (pASD) from parents of typically developing children (pTD). To this end, we performed electroencephalographic recordings of both groups of parents while they had to recognize emotions of face pictures composed of the same or different emotions (happiness or anger) presented in different spatial frequencies. We found no significant differences in the accuracy between groups but lower amplitude modulation in the Late Positive Potential activity in pASD. Source analysis showed a difference in the right posterior part of the superior temporal region that correlated with ASD symptomatology of the child. These results reveal differences in brain processing of recognition of facial emotion in BAP that could be a precursor of ASD.

## Introduction

Attending to and learning from social stimuli is crucial for human lives [1–8]. The neurodevelopmental trajectories of social functioning are closely related to the acquisition of increasingly specialized abilities that enable effective detection of a social agent from birth [2, 5–7, 9, 10]. Among these social abilities, face processing seems to play a key role, since faces entail crucial social information such as other’s identity, emotions, and intentions [3, 5, 8, 11–17].

Neuroimaging and electroencephalographic findings show that facial encoding encompasses diverse subcortical and cortical brain regions and networks, including the fusiform gyrus, the posterior superior temporal sulcus (pSTS) [18–20], the amygdala [21–25], and its interconnections with temporal and frontal regions [23]. Functional magnetic resonance imaging (fMRI) studies have revealed that face perception crucially requires the integration of two subcortical visual pathways [26–28] processing either fine or coarse resolution of the visual signal [28, 29]. On one hand, the magnocellular (M) visual pathway involves the ganglion cells of the retina with a large receptive field and participates in the perception of low-resolution imaging without details (low-spatial frequency, LSF). On the other hand, the parvocellular (P) visual pathway is composed by retinal ganglion cells with small receptive fields and serves to discriminate fine details from a scene and the color (high-spatial frequency, HSF) [27, 30, 31]. These pathways can also be distinguished in the cerebral cortex in early visual areas such as V2 and V3 [32] and in the subsequent cortical pathways [33, 34].

Electroencephalographic (EEG) studies have shown there are at least three known face-sensitive event-related potential (ERP) components: P100 (also known as P1), N170 and N250 [16, 17, 35, 36]. The visual P1 is an early occipital ERP [100 ms after stimulus onset] [36, 37], which is sensitive to low-level properties of the stimulus, such as luminance and contrast [37]. The P1 component also reflects the encoding of coarse characteristics of a face such as position (upright or inverse) [17], displaying a large amplitude for LSF faces in comparison with both HSF [38] and broadband frequency faces [36]. Another early visual ERP associated with face perception is the N170 component (a negativity between 140 ms—200 ms after stimulus onset in the temporo-parietal region) that shows a larger amplitude to faces [17, 35–37, 39, 40]. The N170 shows a greater amplitude for HSF faces than for broadband frequency and LSF faces [36]. Finally, the N250 component is another ERP associated with face perception and is a negative deflection between 250 ms and 300 ms post stimuli in the temporo-parietal brain region [35], which is related to facial identity and visibility [35–37]. The correlation between the N170 and the N250 [35, 37] suggests that, as happens with N170, N250 could encode facial features associated with HSF rather than LSF. Furthermore, not only do these early visual ERPs participate in face processing, but later-stage ERP also contribute to face perception [41–45]. The late positive potential (LPP) has been described as a complex of positive sustained deflections located in the parietal occipital brain region, that occur between 300 ms and 700 ms after stimulus onset, and can also include the P3 component [41, 44, 46]. Interestingly, the evidence has described LPP as larger in response to salient emotional stimuli and to happy faces, when compared to neutral stimuli in typically developing participants [44, 46].

Notably, there are several neurodevelopmental disorders which mainly involve impairments in social stimuli processing and social abilities [8, 47–51]. Studies report that Autism Spectrum Disorder (ASD) affects face perception [4, 52, 53]. However, the ASD clinical heterogeneity and phenotypic diversity makes it difficult to determine the neurobiological mechanisms of these impairments [23, 54–57]. In this context, the study of the endophenotypes of this neurodevelopmental disorder is relevant for linking biological and psychological aspects to a psychiatric phenomenon [58]. Specifically, an endophenotype refers to a heritable feature present in an unaffected family member of an individual who has been diagnosed with a certain medical condition [23, 57]. For ASD, this would be the case of the relatives, of a person with autism, who have no symptoms severe enough (or even have no symptoms at all) to configure a diagnosis of ASD, but share genetic susceptibility. Thus, endophenotypes could suggest neural markers of cerebral processing that are similar to ASD individuals [21, 23–25, 59–61]. Studies also have described endophenotypes as Broader Autism Phenotype (BAP), meaning that symptomatology that may entail social skills, communication traits, and unusual personality features similar to ASD could be present in relatives but be less severe [61–63]. Although behavioral evidence shows conflicting results, the most consistent finding is that individuals with BAP present impairments in the processing of LSF [31, 64]. EEG findings have shown that, compared to individuals with low-level autistic traits, neurotypical adults with high autistic traits describe a lack of P1 modulation by emotion in LSF [38]. Furthermore, it has been reported that the N170 displays a diminished amplitude for faces rather than objects in parents of children with ASD (pASD) compared to parents of typically developing children (pTD) [65]. Regarding the LPP component, evidence has described that individuals with high autistic traits displayed diminished LPP amplitude to faces when compared to non-social stimuli [41, 65].

Considering these findings, our main objective is to disentangle the HSF and LSF processing roles in facial emotion perception and to identify neurobiological markers associated with the differences in this processing as an endophenotype of autism. Therefore, we tested two hypotheses. First, the features of early face-sensitive ERPs and LPP can distinguish emotional processing from HSF and LSF. Secondly, these visual ERP features can differentiate non-symptomatic pASD from pTD. To this end, we assessed the EEG activity evoked by pictures of human faces expressing different emotions by combining happiness and anger emotions with spatial frequencies (i.e., HSF, LSF) in a sample of pTD and pASD. We carried out both a direct test for face-sensitive components (i.e., P1, N170, and N250) and a whole time–scalp analysis specifically looking for later EEG activity (e.g., LPP). According to literature, for early face sensitive components (i.e., P1, N170, N250) we do not expect a specific modulation for emotion stimuli in pTD. In pASD, we expect a decrease in the amplitude not modulated by emotional or spatial frequency for these components. For the LPP component, we expected that 1) salient emotional stimuli (i.e., happy emotion) display greater amplitude, especially when they are presented concurrently in both spatial frequencies. Additionally, we expected that 2) the group of pASD presents a decrease of amplitude modulation of this component given by salient stimuli (i.e., happy emotion).

## Materials and methods

### Ethics statement

All methods and the experimental protocol were approved by the Pontificia Universidad Católica de Chile Ethics Committee and met the principles of the Declaration of Helsinki and the Local Ethical Guidelines for Research Involving Human Subjects. All participants signed a written Informed Consent for their voluntary participation in this study and publication of identifying information/images in an online open-access publication, also approved by the Pontificia Universidad Católica de Chile Ethics Committee. The experiments were carried out at the Laboratorio de Neurociencia Social y Neuromodulación of the Centro de Investigación en Complejidad Social (neuroCICS) of the Universidad del Desarrollo, Santiago, Chile.

### Participants

Forty-three adults participated in the study. The sample comprised biological parents of typically developing children (pTD) and parents of children with ASD (pASD) who had participated in previous research carried out by our team [8]. All parents participated on a voluntary basis and gave written informed consent to participate with their children in the study [8]. The parents participated in a clinical interview to rule out any indicator of psychiatric diagnosis. None of these parents had language impairment, neurological or psychological/psychiatric diagnosis, and none had experienced treatment associated with mental health problems.

Parents of TD children were Spanish speakers, aged from 22 to 42 years (N = 18, 14 women and 4 men, average age 34.61 years, SD = 6.49). Parents having a family member diagnosed with ASD were also excluded from the sample. All children were assessed for alterations in communication and social interaction with the Autism Diagnostic Observation Schedule-2 (ADOS-2) [66]. All children of TD participants (8 boys and 9 girls, average age 3.8 years, SD = 0.5) had to score 2 or less on the ADOS-2 severity scale, which indicates no significant alterations in communication, social interaction, play, or restricted/repetitive behaviors.

Parents of children with ASD were Spanish speakers, aged from 25 to 45 years (N = 25, 17 women and 8 men, average age 36.68 years, SD = 4.85). Children with ASD (14 boys and 6 girls, average age 3.96, SD = 0.57) were selected according to the clinical neurological evaluation following the diagnosis criteria of the Diagnostic and Statistical Manual of Mental Disorders, fifth edition (DSM-5) [67]. All children with ASD met the criteria for both persistent deficits in social communication and social interaction, and the presence of restricted, repetitive patterns of behavior, interests, or activities. Children with ASD were also assessed according to the ADOS-2 severity scale. They scored an average of 5 points, with a low to moderate level of symptoms associated with ASD. Children with a history of auditory processing disorder and/or a syndromic disorder diagnosis were excluded.

There was no significant difference between the two groups of parents on age (Wilcoxon test, p = 0.375) nor sex (p = 0.496).

### Power and sample size

To calculate the minimum sample size and the power of the current study, we used the amplitude of the late potential as the primary outcome. A similar study in broad autism phenotype reports an effect size of f η^2^ = 0.09 [68], which is a large effect [69]. Taking into account the publication bias, and our previous experience in effect size in electrophysiological measure in our experimental setting between clinical and subclinical populations [e.g., 70, 71], we set an intermediated effect size of η^2^ = 0.06. Thus, for the between-within factor interaction in a 2x4 mixed ANOVA with a power of (1-β) = 0.95 and a significant level of α = 0.05, the minimum sample size to find the expected effect was n = 36. Taking this significance effect into account, we recruited participants until both groups of parents had a minimum of 18 participants each. Finally, we recruited in total 43 participants, giving a statistical power of 0.97.

### Experimental design

#### Stimuli

**Fig 1:**
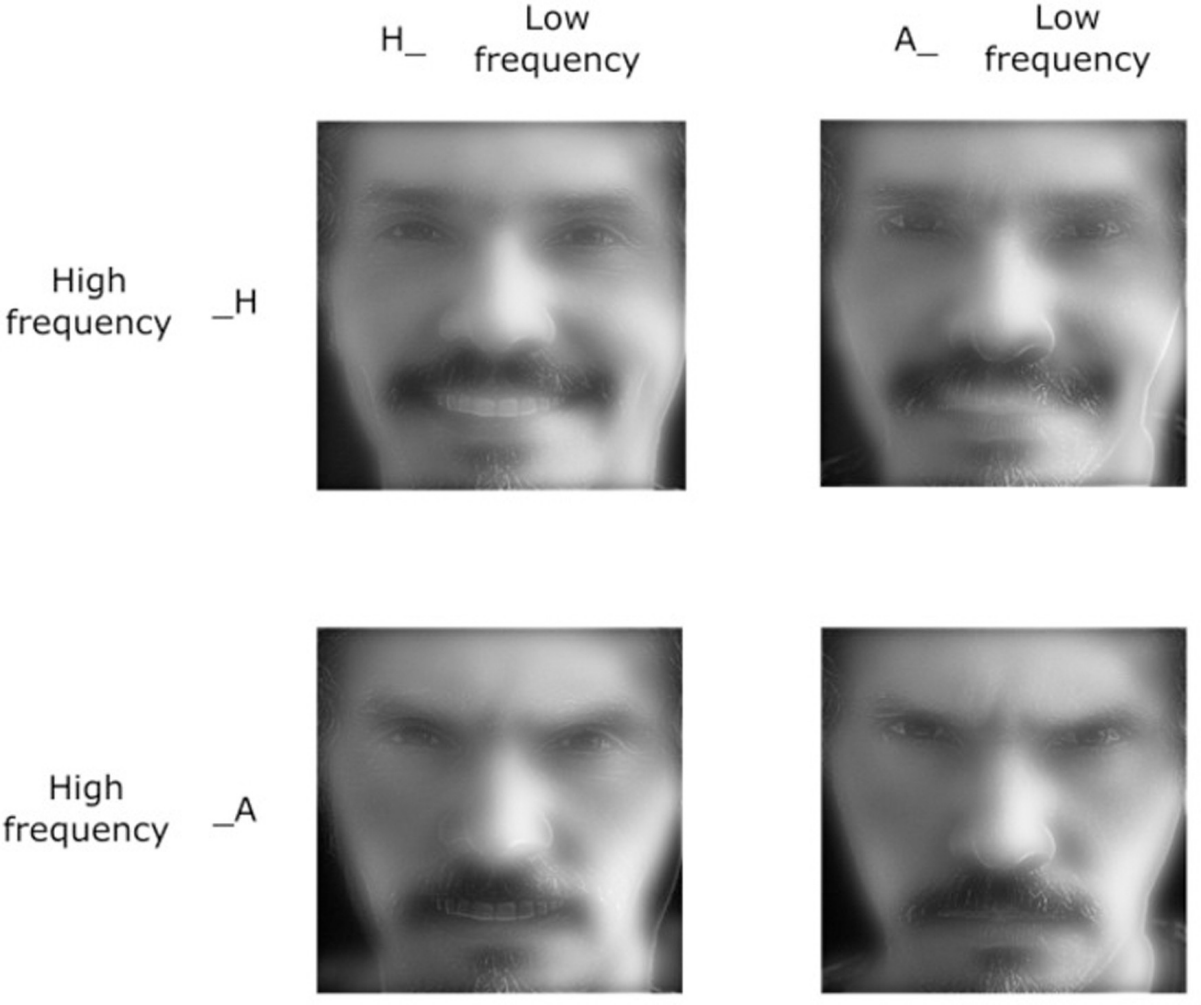
Example of target stimulus used in the experimental design. Target stimuli were hybrid images built by combining a face of the same person composed by HSF with another composed by LSF, expressing congruent or incongruent emotion by combining happiness (H) or anger (A) facial expressions. Target stimuli were based on the Karolinska Directed Emotional Faces standardized database [72], from which 29 identities were selected (14 were pictures of a woman’s face, and 15 were of a man’s face). Thus, a total of 116 different stimuli were presented, with each presented two times in each block of trials. Target stimulus was replaced by a similar, but not identical, to the original image, for illustrative purposes only.

Our target stimuli were hybrid images built by combining two images of the same face: the face of a single person composed of high spatial frequencies (HSFs) with another composed of low spatial frequencies (LSFs) (Fig 1). The emotional expression for each face was manipulated separately. Based on previous studies [27] we used pictures of emotional (happiness and anger) expression, displayed from a frontal point-of-view, from the Karolinska Directed Emotional Faces database [72]. All stimuli used in the current experiment were validated in a pilot study (100 participants) in which happy and angry expressions were matched by intensity. Both expressions were associated with a high recognition rate (over 0.95). These images were desaturated, scaled up to a size of an approximately 5.30° (horizontal) × 6.80° (vertical) visual angle, and filtered using a Butterworth filter to remove either high spatial frequencies (above 24 cycles/face corresponding to about 4 cycles/degree of visual angle) or low spatial frequencies (below 6 cycles/face, corresponding to about 1 cycle/degree). Hybrid stimuli were then created by overlapping one HSF face and one LSF face into a single stimulus (Fig 1). The eyes and mouth position were matched between the LSF and HSF images to obtain a visual overlap yielding the percept of a single face. Note that we did not match faces of different persons and used only images of the same person, and we only manipulated the emotional expression. Thus, we obtained four different stimuli named as follows: "AA", congruent stimulus for anger displayed in both spatial frequencies; "HH", congruent stimulus for happiness; "AH", an incongruent stimulus where anger is presented in LSF and happiness in HSF; and finally, "HA", an incongruent stimulus where happiness is presented in LSF and anger in HSF (Fig 1).

#### Task

**Fig 2:**
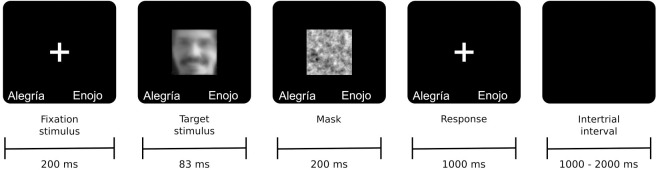
Time outline of a trial. This figure illustrates the time trial task and facial image depicting an incongruent stimulus where anger is presented in LSF and happiness in HSF (i.e., AH). Target stimuli were based on the Karolinska Directed Emotional Faces standardized database [72]. Target stimulus was replaced by a similar, but not identical, to the original image, for illustrative purposes only.

Our task consisted of 232 trials divided into two blocks of 116 trials each (Fig 2). In each trial, a fixation stimulus was displayed for 200 ms. Then, the target stimulus was randomly displayed for 83ms. after which a masked face was presented for 200 ms in order to prevent retinal persistence [e.g., 73, 74]. Next, a black screen was displayed for 1000 ms with the words "Alegría" ("happiness" in Spanish) or "Enojo" ("anger" in Spanish) written in white letters at the bottom. Participants had to choose whether the face expressed happiness or anger by pressing a key on a keyboard using both hands. The hand linked to each choice was random and counterbalanced between the two blocks of trials. A black screen was displayed from 1000 ms to 2000 ms when the trial ended.

Before the experiment began, each participant was trained in the task by using the same structure of the real experiment, but only congruent stimuli were presented. Training blocks consisted of 20 stimuli. In order to facilitate the understanding of the task and only during the training block, a visual feedback on the performance of the participant was presented after each response (an “x” for an error and a ticket for a correct response). Only when the participant obtained an accuracy rate greater than 0.7, the task was considered understood and the experiment began. No feedback was displayed during the real experiment. Correct responses were assessed only in those trials where the stimulus was congruent.

### EEG recordings and analysis

Continuous EEG recordings were obtained with a 64-electrode Geodesic EEG System (Net Station Acquisition). During recordings, the electrodes impedance was kept below 100 kΩ. In the offline analysis, all the electrodes with impedance over 50 kΩ were eliminated and interpolated (none of the recordings present more than 10% of the electrodes above 50 kΩ) [75]. Electrodes were referenced to the Cz electrode during acquisition, and the signal was digitized at 1 kHz. EEG data was then re-referenced offline to the average electrodes and 0.1–30 Hz band-pass filtered. The baseline was chosen from -500 ms to 0 ms, and a time-window from 0 ms to 1500 ms after stimulus onset was analyzed. Artifacts were first automatically detected using a threshold of 150 μV and a power spectrum greater than 2 std. dev. for more than 10% of the frequency spectrum (1 to 30 Hz). Blinking was extracted from the signal by means of ICA. Those trials that included remaining artifacts detected by visual inspection of the signal were eliminated. The mean of artifact-free trials was 268.5 for pTD [246–288] and 272.5 for pASD [237–290] (Wilcoxon test, p = 0.3, n = 45) [8].

The source of the EEG signal was determined by applying a weighted minimum norm estimate inverse solution with unconstrained dipole orientations using the ERP signal for each participant and condition. The individual head model was calculated using a tessellated cortical mesh template derived from a template (MNI152) with 3 x 4000 sources constrained to the segmented cortical surface (three orthogonal sources at each spatial location). To diminish to one dipole per source, the data was reduced by using the norm of the vectorial sum of the three dipoles at each vertex. Since we did not have the individual anatomy to calculate the head model, the spatial precision of the source estimation is insufficient. For minimizing the possibility of erroneous results, the source estimations were calculated and showed only in time windows where we found statistically significant differences at the electrode level, and the resulting source modulation survived multiple comparison correction (cluster-based permutation test < 0.01, cluster threshold detection p < 0.05 and False Discovery Rate, FDR, q < 0.05, applied across the vertices). For visualization purposes, all source results were projected on a high-resolution cortical mesh (~180,000 vertices) [8].

### Statistical analysis

For the purpose of behavioral and ERP analyses, non-parametric tests were used because most of the tested variables did not meet normal distribution. Hence, to compare two means, the Wilcoxon test was used. For comparing variables for several categories, the Friedman signed-rank and the Kruskall-Wallis tests were used. In addition, for response analyses (rate of happiness responses), a mixed ANOVA was conducted in order to estimate the effect of each factor: condition (within factor, with four levels, HH, AA, HA, AH), child’s diagnosis (between factors, with two levels, TD or ASD), and their interaction. Finally, a Mauchly’s Test for sphericity (W) was performed, and corrected p values were calculated for validating the repeated measures analysis of variance ANOVA. Sensitivity index d’ was calculated as d’ = Z (‘‘hit rate”)–Z (‘‘false alarm rate”) [76].

For ERP, we carried out analyses with and without *a priori* hypothesis related to specific time and location (electrodes) of the modulation. Therefore, based on the literature, in our analyses with an *a priori* hypothesis, we extracted the mean of the greater peak of the signal for specific electrodes and time windows. In order to perform a whole time–scalp analysis (that is an analysis without *a priori* hypothesis related to specific time and location of the modulation), we performed a Friedman test (paired) or Kruskal-Wallis (unpaired) test for all electrodes and bins of time, and the obtained results were corrected using a cluster-based permutation test [77]. In this correction, clusters of significant areas were defined by pooling neighboring sites (bins of time-electrode matrices) that showed the same effect (Cluster threshold detection; p < 0.05 for the statistical test carried out in each site, e.g., Friedman test). We obtained permutation distributions by randomly permuting the conditions for each participant (paired test) or permuting the participant for each group (unpaired test), thereby eliminating any systematic difference between the conditions or groups. The same number of trials were used in each permutation as in the original data (for each condition per participant) to control for possible statistical bias due to a different number of trials per condition. After each permutation, the original test (e.g., Friedman test) was computed. After 5000 permutations, we estimated cluster-level significance as the rate of elements of the permutation distribution greater than the observed cluster-level significance. For the source analysis we additionally corrected the results using FDR (q < 0.05) across the vertices. And, for the analysis of the temporal region of interest (ROI) comparing the two groups, we used a mixed linear model using the participant as a grouping factor for random effects and condition as fixed effects (see Eq 1 for details). As this analysis was carried out for each electrode, the results were then corrected using FDR q < 0.05 [8]. For doing the analysis with an *a priori* hypothesis, an amplitude analysis of the P1, N170 and N250 components was performed (P1, maximum voltage between 100 ms and 150 ms in E35 and E39 electrodes ~PO1, PO2; N170 minimum voltage between 160 ms and 200 ms in E27/30 and E44/45 electrodes ~P7, P8; N250 mean amplitude between 250 ms and 350 ms in E27/30 and E44/45 electrodes ~P7, P8) [35, 36]. Finally, we carried out a mixed ANOVA analysis with condition (within factor, with four levels, HH, AA, HA, AH), laterality (within factor, with two levels, left and right) and child’s diagnosis (between factors, with two levels, TD or ASD) as factors.

### Software and data

Behavioral statistical analyses were done in R Software [78], and EEG signal processing was performed in MATLAB using in-house scripts [79, 80], LAN toolbox available at http://neuroCICS.udd.cl/, BrainStorm [81], and OpenMEEG toolboxes [82].

## Results

### 1) Behavioral results

#### 1.a) Parents of TD children

To estimate whether participants correctly understood the task, we assessed the percentage of accuracy when the stimulus was congruent both in happiness and anger conditions (S1 Fig). The group of pTD correctly identified the emotion. Thus, the frequency of correct responses was higher than the number given by chance (accuracy rate 0.67, Wilcoxon test, df = 17, p = 0.002, effect size r = 0.7, global d’ = 0.58, p = 0.002 testing that d’ is other than zero). The group of pTD presents a significant greater accuracy for happiness condition (HH: 0.74; d’ = 1.37; AA: 0.59; d’ = 0.51; differences: df = 17, r = 0.8, p = 2.5e-4). For incongruent stimuli, we assessed whether a spatial frequency bias was shown in HSF or in LSF. We found there was no significant difference between the spatial frequencies (df = 17, p = 0.62, r = 0.1, mean bias 0.03, positive number indicated HSF bias, range [-0.5 0.5], d’ = -2e-17, df = 17, p = 0.7; in the latter case, we used the emotion in the HSF as the signal). For an emotional bias, we found participants tended to choose happiness when the stimuli were incongruent (p = 2.98e-04, r = 0.8, mean bias = 0.084, positive number indicated happiness bias, range [-0.5 0.5]). Nevertheless, this difference was not significant once we compared this with the expected bias given by the difference in the accuracy between happy and angry faces in the congruent conditions and using d’ index assuming “happy” as the signal (mean corrected bias 0.029, df = 17, p = 0.13; r = 0.3, d’ = 0.09, p = 0.08, Table 1). For reaction time, we found a significant modulation per condition (repeated measure ANOVA, *F*_*1*,*17*_ = 5.4, p = 0.32; η^2^ = 0.24) given by a faster response to HH (606.8 ms) than that to AA (644.0 ms) (S2 Fig and S2 Table).

**Table 1:** Behavioral results.

	pTD	pASD	*p-*value
			(< 0.05)
**BEHAVIORAL RESULTS**			
**Mean global accuracy rate**	0.67 (SE = 0.04)	0.68 (SE = 0.03)	1
(when stimulus is congruent in both emotions).	p = 0.002*	p = 1.43e-4*	
**Mean happiness accuracy rate**	0.74 (SE = 0.04)	0.77 (SE = 0.04)	0.68
(when stimulus is congruent in happiness emotion).	p = 8.51e-4*	p = 3.87e-5*	
**Mean anger accuracy rate**	0.59 (SE = 0.04)	0.59 (SE = 0.04)	0.87
(when stimulus is congruent in anger emotion).	p = 0.03*	p = 0.047	
**Mean happiness accuracy rate—Mean anger accuracy rate**	p = 2.49e-4*	p = 3.72e-5*	0.37
**Mean spatial frequency bias** ^(^ ^#^ ^)^	0.03 (SE = 0.04)	0.03 (SE = 0.03)	0.81
(when stimulus is incongruent).	p = 0.62	p = 0.29	
**Mean uncorrected emotional bias** ^(##)^	0.08 (SE = 0.01)	0.11 (SE = 0.02)	0.47
	p = 2.98e-4*	p = 8.97e-5*	
**Mean corrected emotional bias** ^(^ ^##^ ^)^	0.0292	0.0402	0.84
	p = 0.13	p = 0.025*	

Abbreviations: DS = standard deviation; SE = standard error; Mean = proportion (percentage);

^(^*^)^ Statistically significant difference (p < 0.05 Wilcoxon test);

^(#)^ Mean spatial frequency bias: positive mean = high frequency bias; negative mean = low frequency bias;

^(##)^ Mean (uncorrected and corrected) emotional bias: positive mean = happy bias; negative mean = angry bias.

#### 1.b) Parents of children with ASD and comparisons between the two groups of parents

**Fig 3:**
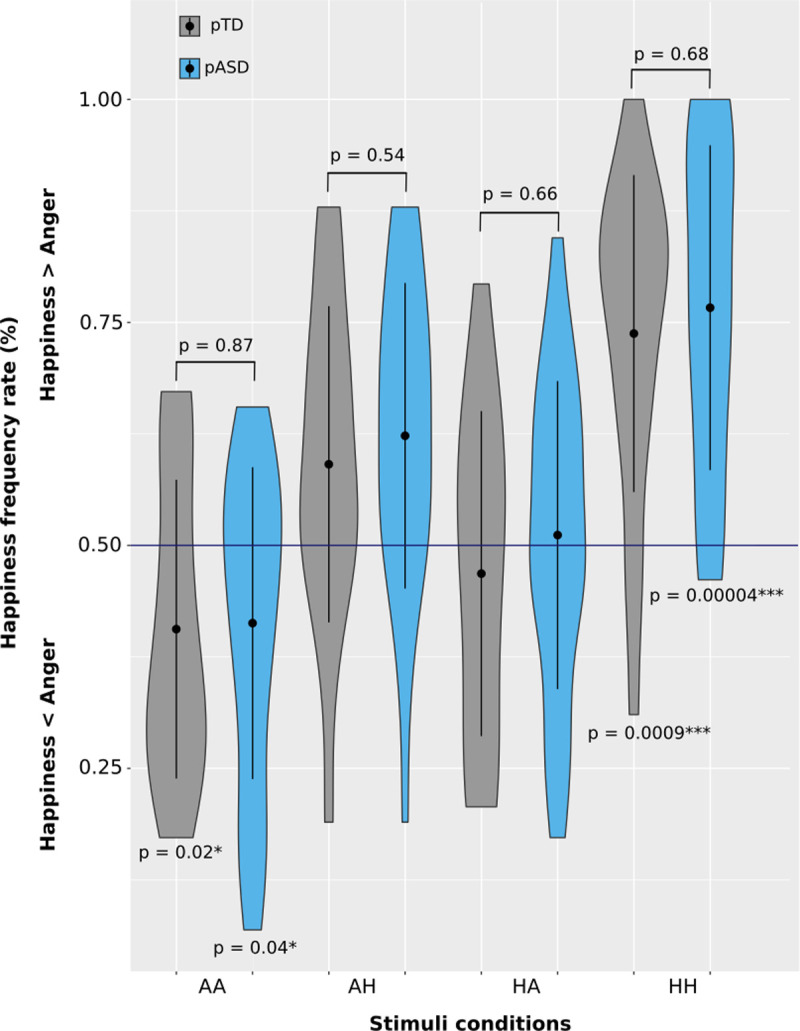
Happiness frequency rate for each stimulus condition in both groups of parents. Frequency distribution of happiness rate responses in all stimuli conditions in pTD and pASD. Black point shows the mean of the distribution. Bars indicate standard deviation. Happiness frequency rate responses above 0.5 = * < 0.05; ** < 0.01; *** < 0.001.

The group of pASD demonstrated a similar accuracy performance to pTD (df = 24, mean accuracy rate: 0.68, p = 1.4e-4, r = 0.82, d’ = 0.64, p = 0.0001, happiness accuracy rate: 0.77, r = 0.84, d’ = 1.8, p = 3.8e-5, anger accuracy rate: 0.59, r = 0.39, d’ = 0.56, p = 0.047, comparison between the pTD—pASD groups, unpaired Wilcoxon test, df = 41, p > 0.68, see also mixed ANOVA below). Accuracy rate was greater for happiness (df = 24, r = 0.82, p = 3.7e-5), with no differences with the control group (comparison between the pTD—pASD groups: df = 41, r = 0.18, p = 0.37). As the pTD in incongruent stimuli, pASD showed no significant bias for spatial frequency (p = 0.29, mean bias 0.04, d’ = -3e-17, comparison between the pTD—pASD groups: p = 0.8), but they showed a significant bias for happiness (corrected mean bias 0.04, p = 0.025, comparison between the pTD—pASD groups: p = 0.8, Table 1; d’ = 0.22, p = 0.011). In addition, no significant differences were found between both groups of parents for incongruent conditions, when testing the rate of happiness responses (unpaired Wilcoxon test, df = 42, p > 0.54, Fig 3; happiness frequency rate responses other than 0.5: AA pTD p = 0.02; AA pASD p = 0.047; HH pTD p = 0.0009; HH pTEA p = 0.00004).

Additionally, for testing differences between groups, a mixed ANOVA was conducted with the aim of revealing whether both the effect of the stimuli condition and the diagnosis of the child were associated with the frequency rate of happiness (for details see S1 Table). For diagnosis of the child (TD or ASD), no significant effect was found (*F*_*1*,*41*_ *=* 1.5; p *=* 0.21; η^2^ = 0.006). Instead, a significant effect of the stimuli condition was observed (*F*_*3*,*123*_ *=* 27.8; p *=* 7.97e-14; η^2^ = 0.362; sphericity correction p = 3.8e-10) indicated a greater correct response rate for HH condition. Also, no significant effect was found for the interaction between both factors (*F*_*3*,*123*_ *=* 0.07; p *=* 0.97; η^2^ = 0.001; S1 Table).

For reaction time, we found a significant modulation per condition (repeated measure ANOVA, *F*_*3*,*72*_ = 5.5, p = 0.001; η^2^ = 0.01) given by a faster response to HH (652.8 ms) than all other conditions (AA: 700.1 ms, AH: 707 ms, HA: 689.6 ms). No differences were found between groups (Mixed ANOVA, Diagnosis: *F*_*1*,*41*_ = 1, p = 0.3; η^2^ = 0.02 interaction Diagnosis and Condition *F*_*3*,*123*_ = 0.3, p = 0.7, η^2^ = 0.0004) (S2 Fig and S2 Table).

### 2) ERP and brain source results

#### 2.a) Early face-sensitive ERP components

**Fig 4:**
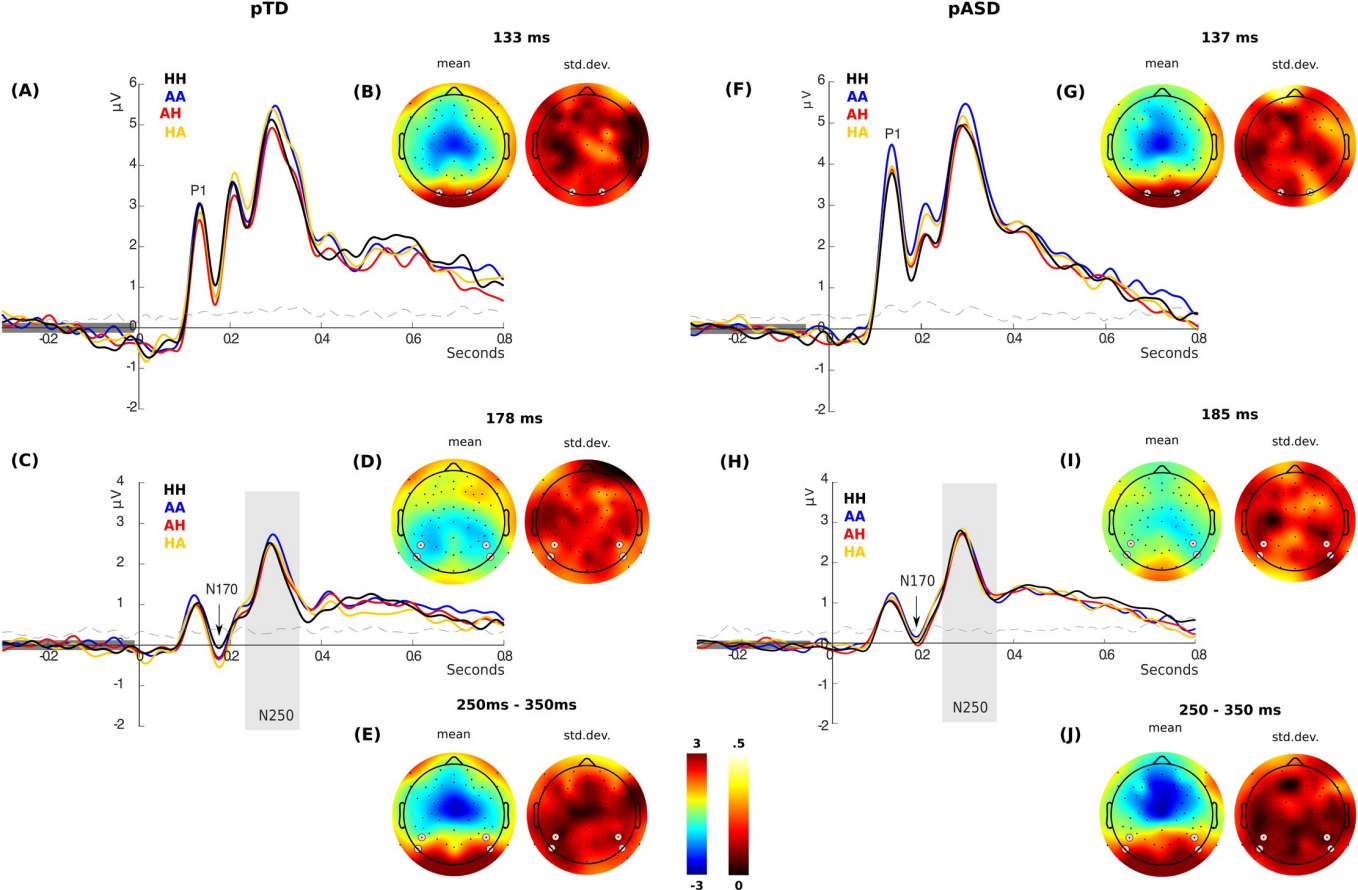
Face-sensitive ERP components in the two groups of parents. **(A)** P1 amplitude of all four conditions of the experiment in pTD and **(F)** in pASD from the electrodes highlighted in white circles in the topographic plots (B and G). **(B)** Topographic distribution of both mean and standard deviation among conditions for the activity at the ERP peak as shown in (A). **(G)** Topographic distribution of both mean and standard deviation among conditions for the activity at the ERP peak as shown in (F). **(C)** N170 (black arrow) and N250 (gray box) amplitude of all four conditions of the experiment in pTD and **(H)** in pASD from the electrodes highlighted in white circles in the topographic plots (D, E, I and J). **(D)** Topographic distribution of both mean and standard deviation among conditions for the activity at the ERP peak as shown in (C). **(E)** N250 amplitude of all four conditions of the experiment in pTD between 250 ms and 350 ms as shown in (C). **(I)** Topographic distribution of both mean and standard deviation among conditions for the activity at the ERP peak as shown in (H). **(J)** N250 amplitude of all four conditions of the experiment between 250 ms and 350 ms as shown in (H). The black line shows the "HH" condition (congruent stimulus for happiness), blue line the "AA" condition (congruent stimulus for anger), red line the "AH" condition (anger in LSF and happiness in HSF), and orange line the "HA" condition (happiness is in LSF and anger in HSF). Dash line shows the variance among conditions.

Using a prior knowledge of the time and electrodes of the modulation based on previous findings [35, 36], we first performed an amplitude analysis of the P1, N170 and N250 components (Fig 4).

*P1 component*. Fig 4 shows the amplitude of all four conditions of the experiment in pTD (Fig 4A) and in pASD (Fig 4F). Fig 4 also shows the topographic distribution of both mean and standard deviation among conditions for the activity at the P1 peak in pTD (Fig 4B) and in pASD (Fig 4G). Mixed ANOVA results did not show significant differences between the two groups of parents neither in condition (Group x Condition, P1 amplitude: *F*_*3*,*123*_ *=* 0.6; p *=* 0.5; η^2^ = 0.001), nor laterality (Group x Electrode, P1 amplitude: *F*_*1*,*41*_ *=* 1.3; p *=* 0.2; η^2^ = 0.002), nor even in the interactions between factors (Group x Condition x Electrode, P1 amplitude: *F*_*3*,*123*_ *=* 0.4; p *=* 0.6; η^2^ = 0.0002). However, the interaction between condition and laterality (Condition x Electrode, *F*_*3*,*123*_ = 2.7, p = 0.047; η^2^ = 0.001) were significant for P1 amplitude. Post-hoc analysis showed that there was a significant difference between the P1 amplitude in AA and AH conditions (p *=* 0.016, Bonferroni corrected) given by differences in the right electrodes (p *=* 0.01, Bonferroni corrected).

*N170 component*. The amplitude of N170 in all four conditions of the experiment in pTD (Fig 4C) and in pASD (Fig 4H) is shown in Fig 4. Fig 4 also shows the topographic distribution of both mean and standard deviation among conditions for the activity at the N170 peak in pTD (Fig 4D) and in pASD (Fig 4I). Mixed ANOVA results did not show significant differences between the two groups of parents neither in condition (Group x Condition, N170 amplitude: *F*_*3*,*123*_ *=* 2.1; p *=* 0.1; η^2^ = 0.003), nor laterality (Group x Electrode, N170 amplitude: *F*_*1*,*41*_ *=* 0.3; p *=* 0.5; η^2^ = 0.002), nor even in the interactions between factors (Group x Condition x Electrode, N170 amplitude: *F*_*3*,*123*_ = 0.9; p *=* 0.4; η^2^ = 0.001).

*N250 component*. Fig 4 shows the amplitude of all four conditions of the experiment in pTD (Fig 4C) and in pASD (Fig 4D). Fig 4 also shows the topographic distribution of both mean and standard deviation among conditions for the activity at the N250 peak in pTD (Fig 4E) and in pASD (Fig 4J). Mixed ANOVA results did not show significant differences between the two groups of parents neither in condition (Group x Condition, N250 amplitude: *F*_*3*,*123*_ *=* 0.4; p *=* 0.7; η^2^ = 0.0003), nor laterality (Group x Electrode, N250 amplitude: *F*_*1*,*41*_ *=* 0.5; p *=* 0.4; η^2^ = 0.005), nor even in the interactions between factors (Group x Condition x Electrode N250 amplitude: *F*_*3*,*123*_ *=* 0.4; p *=* 0.6; η^2^ = 0.0003).

#### 2.b) Whole time-scalp ERP analysis

**Fig 5:**
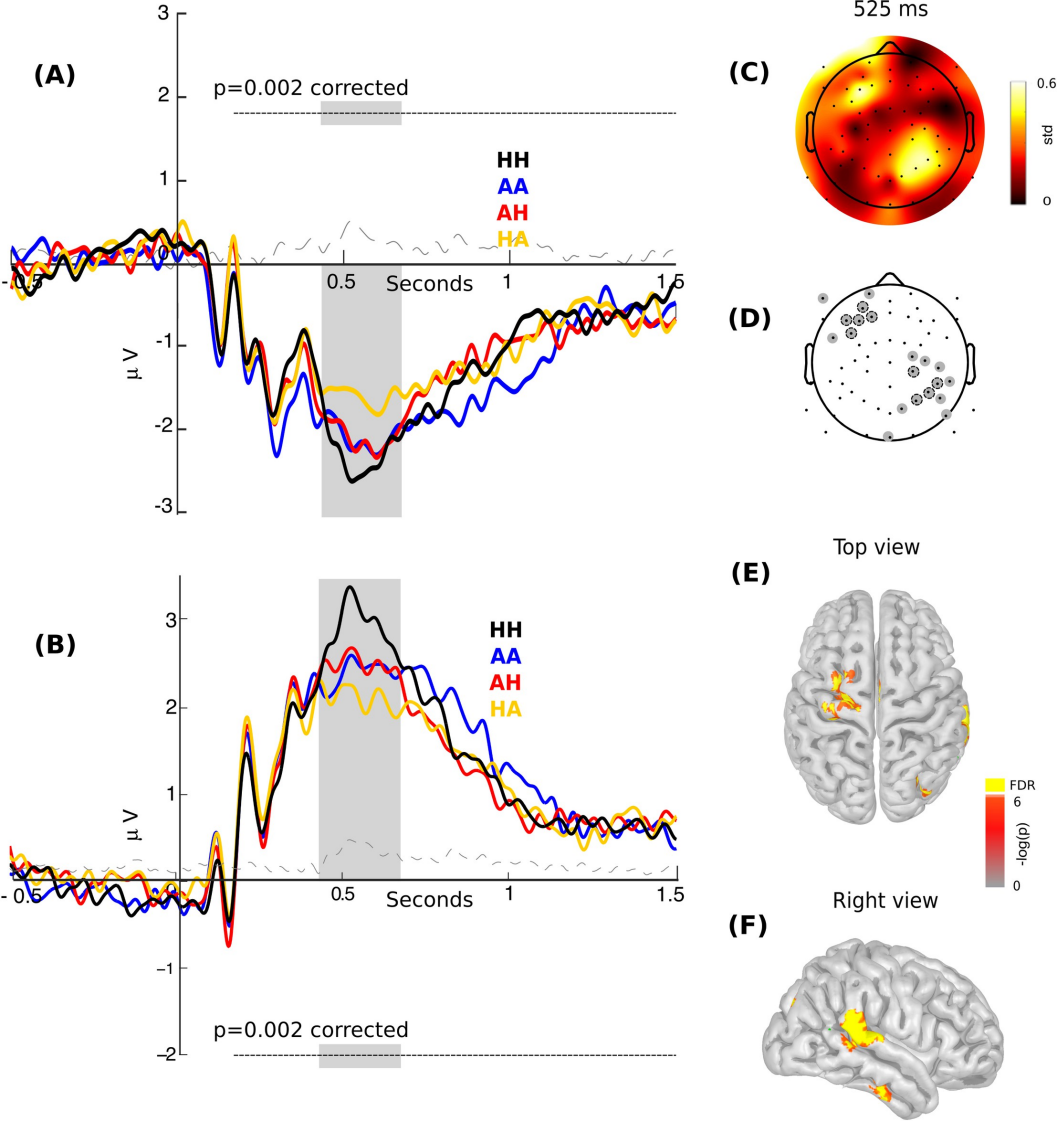
Electrical brain modulation among all conditions for pTD. **(A)** ERPs of all four conditions of the experiment extracted from left frontal and **(B)** right parietal electrodes. The gray region indicates a significant area (Cluster based permutation test, corrected p = 0.001, critical alpha for four contrasts = 0.0125). The black line shows the "HH" condition (congruent stimulus for happiness), blue line the "AA" condition (congruent stimulus for anger), red line the "AH" condition anger in LSF and happiness in HSF), and orange line the "HA" condition happiness is in LSF and anger in HSF). Dash line shows the variance among conditions. **(C)** Topographic distribution of the activity at the 525ms ERP peak as shown in (A). **(D)** Topographic distribution of the ROI of electrodes. The gray circles are showing ROI and gray circles with dotted black lines are showing significant electrodes. **(E and F)** Estimated sources of the contrast for all four conditions in the pTD at the 525 ms peak, as shown in (A), (B) and (C) (red: CBP test < 0.0125, CTD Friedman test p < 0.05, and yellow: FDR q < 0.05).

*Parents of TD children*. Since the later ERPs are more variable and are more sensitive to specific task demand, we additionally explored brain modulation evoked by the stimuli in all possible combinations of spatial frequency and expressed emotion without any *a priori* hypothesis related to the time or the electrodes of possible modulation. Thus, using a whole time–scalp analysis and cluster correction (see Materials and Methods section), we found significant brain modulation among all basic conditions (i.e., AA, AH, HA, HH) between 430 ms and 730 ms after the stimuli presentation in a cluster of electrodes in the left frontal and right parietal region (Cluster-based permutation [CBP] test, p = 0.001, Cluster threshold detection [CTD] Friedman test p < 0.05, peak at 525 ms, Fig 5A and 5B). Source analysis showed modulation in the dorsolateral prefrontal cortex (Fig 5E; FDR q < 0.05) and a right lateralized activity in the posterior part of the superior temporal region (Fig 5F; FDR q < 0.05) during the peak of the scalp modulation at 525 ms. As this peak occurs just before the mean of participant response, we correlated the mean of this component with reaction time. This correlation was not significant, indicating that this component cannot be due to motor responses (Spearman n = 18, rho = - 0.08, p = 0.46).

**Fig 6:**
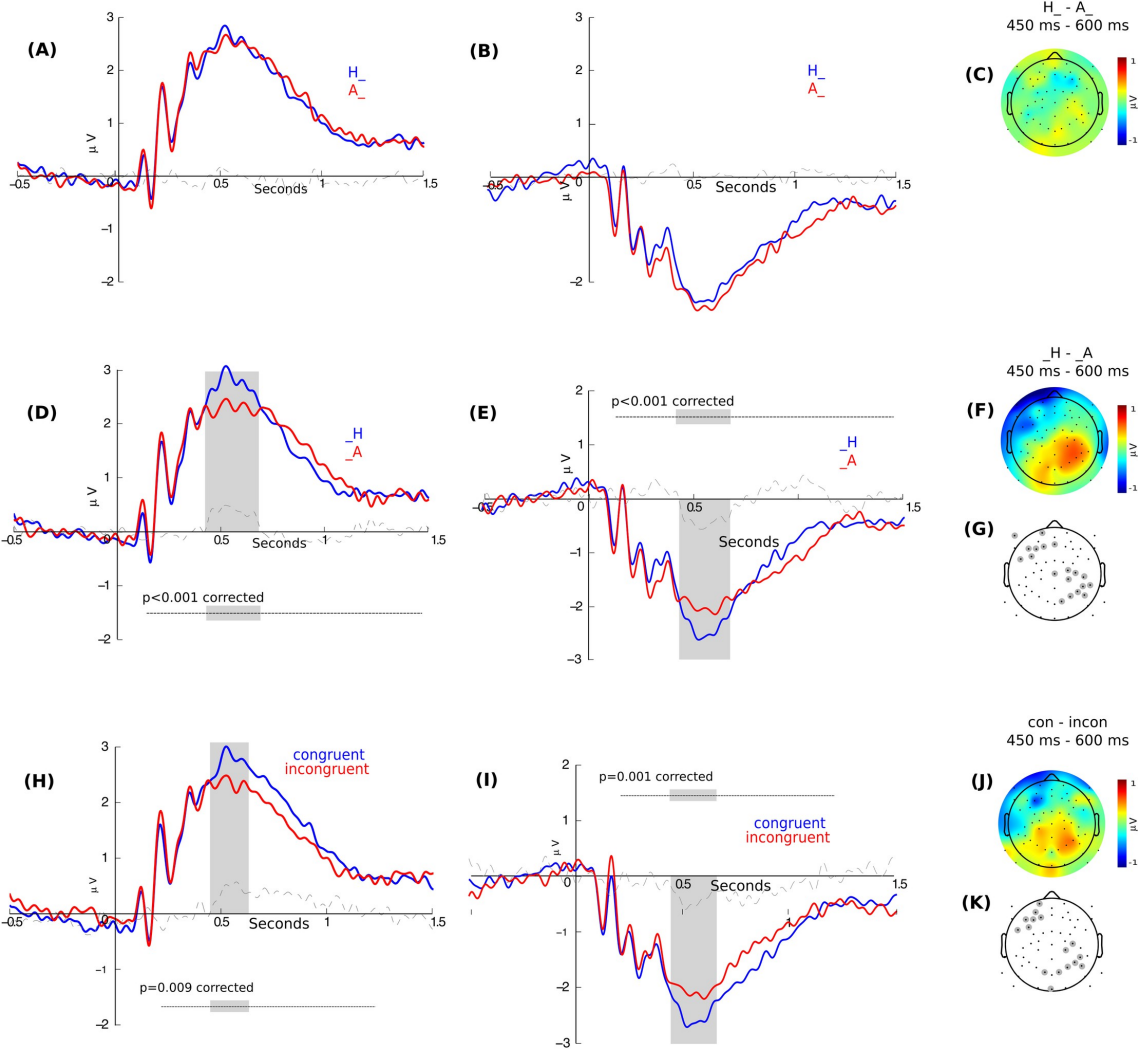
Right parietal and left frontal ERP modulation in each stimuli condition in pTD. **(A)** ERP modulation by LSF extracted from right parietal and **(B)** left frontal electrodes. The blue line shows happiness and the red one anger (i.e., H_ and A_). **(C)** Topographic distribution of the difference between conditions as shown in **(A and B)**. **(D)** ERP modulation by HSF extracted from right parietal and **(E)** left frontal electrodes. The blue line shows happiness and the red one anger (i.e., _H and _A). The gray region indicates a significant area (Cluster based permutation test). **(F and G)** Topographic distribution of the difference between conditions as shown in (D and E) and cluster of significant electrodes at the indicated time. **(H)** ERP modulation in congruent and incongruent conditions extracted from right parietal and **(I)** left frontal electrodes. The blue line shows congruent condition and the red line incongruent condition. The gray region indicates a significant area (Cluster based permutation test). **(J and K)** Topographic distribution of the difference between conditions as shown in (H and I) and cluster of significant electrodes at the indicated time. The dash line shows the difference between conditions.

Given these results, we explored a possible modulation given by different stimuli features by means of categorical analyses. Specifically, we assessed for modulations given by the emotion in the LSF irrespective of the emotion in HSF (i.e.: "H_" = happiness in LSF; "A_" = anger in LSF), modulations given by the emotion in the HSF irrespective of the emotion in the LSF (i.e., "_H" = happiness in HSF, "_A" = anger in LSF) and, finally, modulations given by the presentation of congruent emotion in both frequencies (Fig 6). There was no significant difference between H_ and A_ conditions (Fig 6A–6C). On the contrary, we found a significant difference between _H and _A conditions in a left frontal and posterior cluster of electrodes between 425ms and 685ms (CBP test p < 0.001, CTD Wilcoxon test p = 0.05) (Fig 6D–6G). The difference is associated with a greater amplitude for happiness emotion when it is displayed in HSF. Finally, we found a significant difference between the congruent and incongruent conditions irrespective of the emotion (CBP test p = 0.009, CTD Wilcoxon test p = 0.05) (Fig 6H–6K). The topographic distribution evidenced a left frontal and posterior group of electrodes as the main contributors to this brain modulation between 450ms and 630 ms.

**Fig 7:**
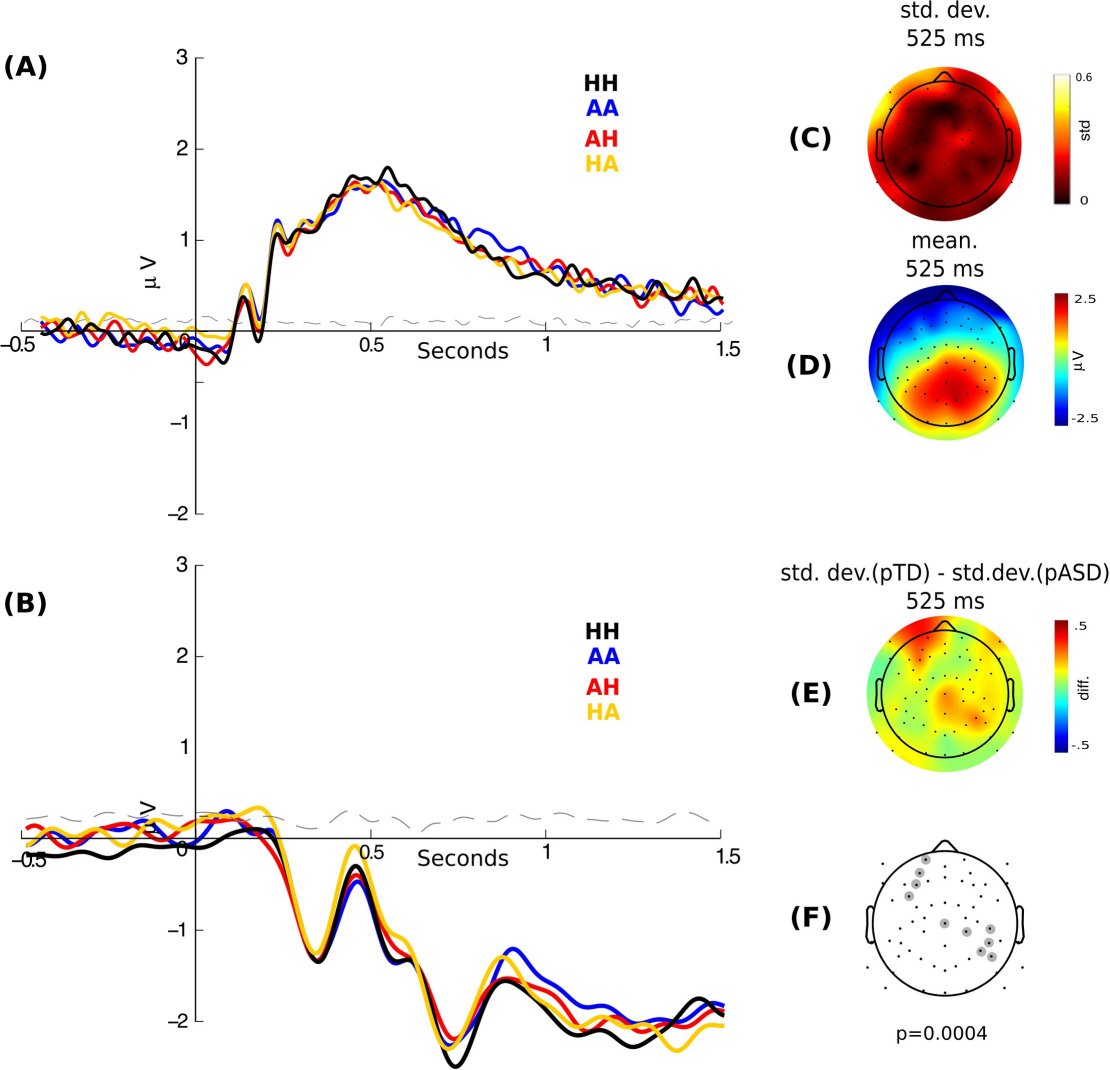
Electrical brain modulation among all conditions for pASD and pTD comparison. **(A)** ERPs of all four conditions of the experiment extracted from right parietal and **(B)** left frontal electrodes. There was no significant difference among conditions (Cluster based permutation test, p > 0.05; ROI analysis, right parietal electrode, 450–630 ms, as the result in Fig 3, Freidman p = 0.14). The black line shows the "HH" condition (congruent stimulus for happiness), the blue line the "AA" condition (congruent stimulus for anger), the red line the "AH" condition anger in LSF and happiness in HSF), and the orange line the "HA" condition happiness is in LSF and anger in HSF). **(C)** Topographic distribution of standard deviation among conditions at the ERP peak of 525ms as shown in (A and B). **(D)** Topographic distribution of mean among conditions at 525ms. **(E)** Topographic distribution of the difference of the standard deviation among conditions for pTD and pASD groups at 525ms. **(E)** Topographic distribution of the electrode at 525 ms of the significant cluster of the difference of the standard deviation among conditions between pTD and pASD groups (Cluster based permutation test, CTD p = 0.05 Wilcoxon test, corrected p = 0.0004).

*Parents of children with ASD and comparisons between the two groups of parents*. We first carried out a whole time-scalp analysis for differences in the LPP in pTD. Interestingly, cluster-based permutation tests showed no significant difference among the conditions in the significant cluster of electrodes observed in pTD (Fig 7). In order to compare both groups of parents, we compared the mean of all conditions between groups indicating the main effect of the group, and we did not find significant modulations. Finally, we compared the variance among conditions between groups, exploring the interaction between groups and conditions, and we found a significant difference between groups (CBP test, p = 0.004, CDT Wilcoxon p = 0.05, Fig 7). As in the pTD group, the mean amplitude of this component por pASD group did not correlate with reaction time (n = 25, Spearman rho = - 0.02, p = 0.8).

To test the factors underlying the main differences between groups and based on the results obtained from pTD (Figs 5 and 6), we explored differences in brain modulation between groups in the time of the main modulation using multiple mixed linear models. We fitted a mixed linear model that included a regressor for each emotion presented in each spatial frequency and interaction between the emotions present in difference frequency in a temporal ROI where the main modulation in pTD was observed. This model is depicted in the following Eq (1):

$$\mu \text{V}=\text{int}+{\text{Emo}}_{\text{LSF}}+{\text{Emo}}_{\text{HSF}}+{\text{Emo}}_{\text{LSF}*\text{HSF}}+\text{pASD}+\text{pASD}*\left({\text{Emo}}_{\text{LSF}}\right)+\text{pASD}*\left({\text{Emo}}_{\text{HSF}}\right)+\text{pASD}*\left({\text{Emo}}_{\text{LSF}*\text{HSF}}\right)+\text{Sex}$$ (1)

**Fig 8:**
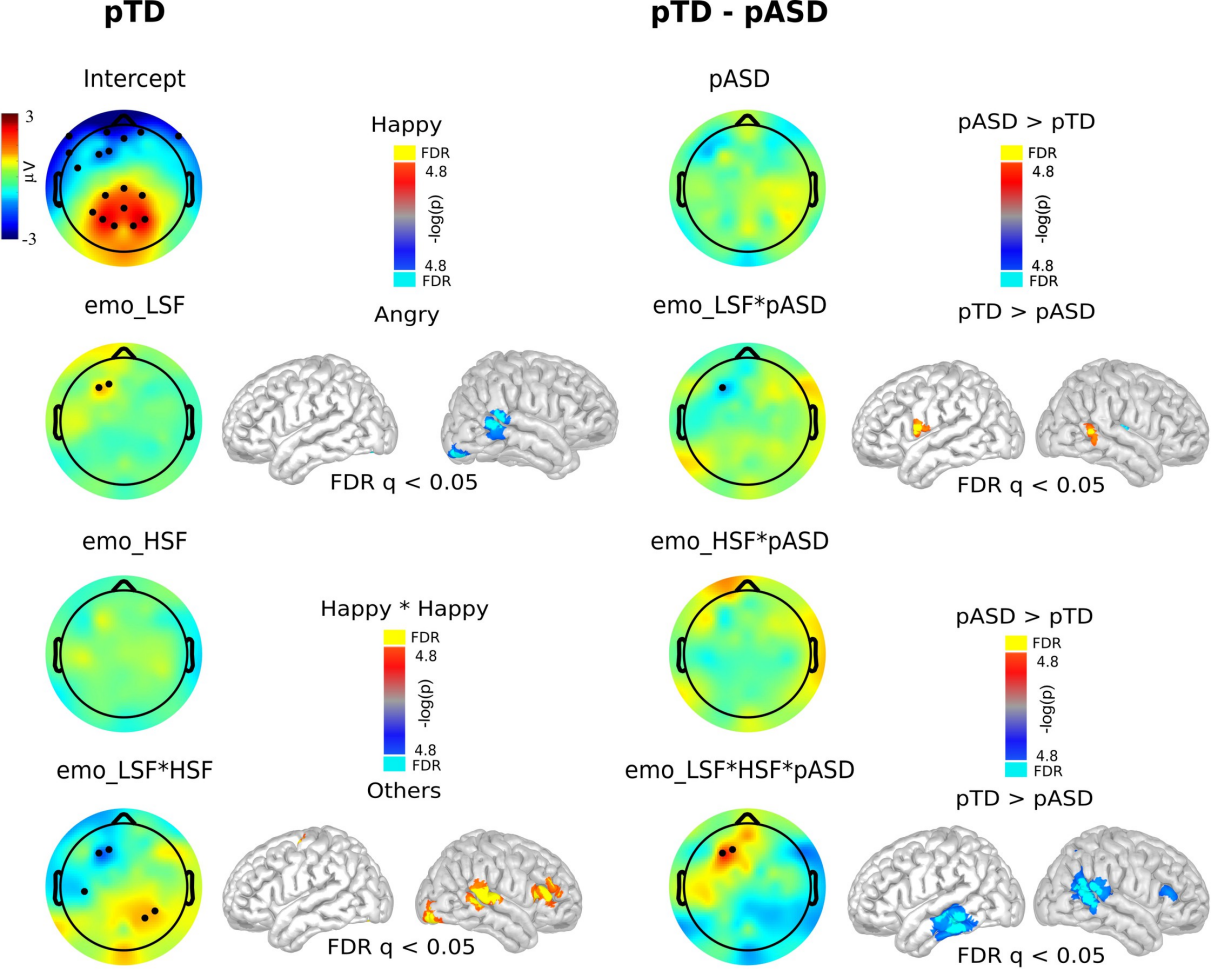
Scalp distribution and sources of frequency, frequency interaction and group regressors. From left to right, the first column shows brain modulation in pTD and the third column shows the difference between both groups of parents. The black circles indicate electrodes that show statistical modulation (FDR q < 0.05). The second and fourth columns show the brain sources of the modulation when we found significant findings in the scalp (FDR q < 0.05) in pTD (first column) and between the two groups of parents (third column), respectively.

In this model, happiness was set to one. Interestingly, the main modulation was found for Emo_LSF*HSF_ interaction, with no significant differences for *Emo*_*HSF*_. As these results indicate, the positive emotion expression presented congruently in both spatial frequencies generated the greatest brain responses, and this brain response is selectively affected in pASD (Fig 8, second and bottom line). Additionally, pTD showed a modulation for Emo_LSF_. Interestingly, pASD evidenced a decrease in this modulation (Fig 8, second line, Emo_LSF*pASD_). While in the HSF a non-significant difference between the two groups was observed (Fig 8, third line), the difference between the integration between spatial frequencies (as indicated by Emo_LSF*HSF*pASD_ regressor) showed a significant difference between the two groups of parents (Fig 8, bottom line). Source analysis in pTD for Emo_LSF*HSF_ regressors showed a right modulation in the occipital region, posterior part of superior and middle temporal gyrus and inferior frontal gyrus as the main brain regions contributing to the integration between the two spatial frequencies (Fig 8, bottom line, right row). Additionally, for Emo_LSF_ a right modulation was found in the occipital region, posterior part of superior and middle temporal gyrus and inferior frontal gyrus. For the comparison between groups, the source analysis showed a decrease in the modulation of the temporal brain region in pASD (Fig 8, bottom line, left row). Specifically, for the Emo_LSF*HSF*pASD_ regressor, a negative modulation in the right superior and middle temporal gyrus and inferior frontal gyrus was found together with negative modulation in the left middle and inferior temporal gyrus. Additionally, for the EmoLSF_*pASD_ regressor we found a positive modulation in the right posterior middle temporal gyrus and left inferior frontal gyrus. Taken together, these results indicate that pASD could display an alteration in the integration between M and P pathways for the processing of visual perception of socially salient stimuli in spite of not evident behavioral differences (accuracy and symptoms).

Finally, we tested whether the difference in brain activation between both groups of parents have a relationship to the possible genetic load of the pASD. For this, we extracted the source activation in a posterior temporal region (region that survived FDR in pTD group, regressor EMO_LSF*HSF_) for the pASD group during HH condition and correlated this with the ADOS score of their children (as a raw proxy of genetic load) and accuracy in HH condition. Interestingly, this activation correlated negatively with the severity of ASD symptomatology (rho = - 0.51, p = 0.007, n = 25) but not with the accuracy (rho = 0.29, p = 0.14; partial correlation ERP-ADOS: rho = - 0.49, p = 0.01; ERP-Accuracy: rho = 0.24, p = 0.25, n = 25).

## Discussion

Due to its clinical heterogeneity and phenotypic variability, determining the underlying neurobiological mechanisms of ASD has become a challenging goal [23, 54–57]. Since it is well known that ASD affects face perception and recognition, we tested whether these impairments are associated with problems in the processing of HSF and LSF. As ASD has a strong genetic basis, these impairments could be inherited traits, thus the study of brain processing in relatives of children with ASD diagnosis (in this case, their biological parents) would help to identify biological markers and possible mechanisms of the disease [21, 23–25, 59, 60]. In this context, our research contributes to disentangling the brain processing of emotional stimuli present in ASD. Specifically, our experimental paradigm dissociated emotional brain processing coming predominantly not only from HSF and LSF of the visual stimuli, but also from processing that requires the integration of both. Taking into consideration that suboptimal stimuli presentations might be processed differentially by the P and M visual pathways, and that the P pathway might process brief stimuli more efficiently, our design considered a brief time of target stimuli displayed (83 ms) in order to induce a predominant fast response to emotional stimuli [83].

Our findings demonstrate that, in pTD, congruent happiness stimuli generated the greatest brain response. These responses could be related with social value in terms of social reward generating a greater saliency [84–86]. Indeed, ASD participants present an abnormal pupillary response to happy faces [87]. Interestingly, it seems that for this effect, integration of both HSF and LSF visual pathways is required. Coincidentally, findings indicate that brain regions related to the integration of the two visual frequencies are crucial when it comes to understanding complex cognitive and emotional processing as perception of human faces [15, 87–90]. Notably, no significant differences were found between the two groups of parents in early face-sensitive ERP components (Fig 4). These results could indicate that early ERPs are encoding coarse characteristics of a face such as position (upright or inverse) [17, 37] and basic discrimination. This could be related to both groups showing similar behavioral performance, which can be interpreted as a compensatory strategy developed by pASD for discriminating emotion, as proposed for other disorders [71]. However, the LPP component in the HH condition correlated with the children’s symptomatology (i.e., ADOS-2 score), but not with the accuracy. In accordance with previous evidence [44, 46], the difference found between the two groups of parents in the LPP might be reflecting an attentional response to salient emotional stimuli, such as happy faces, and not necessarily any emotional discrimination. Moreover, our brain source analysis showed that the pSTS and the occipital region (Figs 5E and 8 bottom line) are involved in this integrative processing in pTD. It is well-known that the STS has been described not only as part of the processing network for facial expressions, but also as a hub for social perception and cognition [18, 19]. Another brain region involved in this integration is the IFG, since the IFG is active during the response to emotional facial expressions in neurotypical participants [15, 87–90]. This evidence, together with the use of emotional faces stimuli in our experimental paradigm highlights the notion that both face and emotional processing are part of a brain network functioning in a close integration with the social cognitive network.

Of note, and as we observed in a previous comparative study between children with and without autism [8], and in accord with previous findings [91], results showed no significant differences between the two groups of parents. However, unlike the case of pTD, pASD did not demonstrate an LPP saliency for congruent happiness stimuli. This result could indicate a weaker saliency response reflected in a diminished emotional brain modulation in pASD. This weaker response could be related to either a decrease or an alteration in the neuronal mechanism that integrates the information coming from different spatial frequencies. Moreover, neural responses in STS are elicited by changes in head/gaze direction and also in emotional expression [92]. Thus, our results may indicate that perception of emotional facial expressions depends on a neural network that entails the perception of changeable features of the human face that are crucial for social communication [19, 91]. The decrease in the modulation of the temporal brain region observed in pASD (Fig 8, bottom line, left row) may reveal that differences between ASD and neurotypical development in cortical processing of faces expressing emotion could be broader and more complex than a mere consequence of social difficulties, and should receive more attention in future research [23, 27, 31, 61–63]. Indeed, the activation of this brain area in pASD correlates negatively with the symptomatology of their children (in terms of ADOS-2 score) and may reflect the genetic features that ASD entails.

Several limitations to this study include the small sample size, which should be considered when interpreting the findings. Also, although pASD were selected based on psychiatry criteria (i.e., a family member of an individual who has been diagnosed with a certain medical condition [57], standardizing diagnosis instruments such as BAP-Q [93], ADOS-2 [66] or ADI-R [94] could be valuable for BAP measurement. Such scales could be useful for better discrimination between BAP and a possible ASD that could have remained undetected. Moreover, considering evidence that autistic-like traits could be also found in any typical population [95–97], the consideration of scales such as the AQ [98] in studies of non-clinical groups could contribute to better comprehension of research results such as those described here.

On the other hand, we did not incorporate neutral stimuli (such as neutral face, landscapes, or body parts), thus we are unable to determine whether anger is less salient by itself or whether it is less salient than happiness. Furthermore, we are also unable to determine whether there is any difference in the perception of spatial frequencies by using standard stimuli such as Gabor or sinusoidal gratings [47, 64, 98–102]. We tackled these problems by using the multiple mixed linear models, which showed a correlation between frequency and emotion. Although our EEG results met statistical criteria, these are exploratory results that should be replicated in an independent sample.

Overall, our findings show a possible neural mechanism involved in the differences in the emotional processing between ASD and neurotypical development. This evidence could contribute toward clarifying why social functioning could be highly demanding and often very difficult to manage for autistic or autistic-like persons. The evidence here could also help clinicians and researchers to better understand this neurodevelopmental condition, and thus to identify risk factors and to provide timely diagnosis and treatment.

## Supporting information

S1 Fig Boxplot of frequency rate of happiness in all stimuli conditions in each group of parents. Four different stimuli were named as it follows: "AA", congruent stimulus for anger in which is displayed in both spatial frequencies; "HH", congruent stimulus for happiness; "AH", an incongruent stimulus where anger is presented in LSF and happiness in HSF; and finally, "HA", an incongruent stimulus where happiness is presented in LSF and anger in HSF.
(TIF)

S2 Fig Reaction time distribution for each stimulus condition in both groups of parents. Reaction time for the subject’s choice of emotional stimuli in all stimuli conditions in pTD and pASD. Black point shows the mean of the distribution. Bars indicate standard deviation.
(TIF)

S1 Table Mixed ANOVA and sphericity test of frequency rate of happiness in all stimuli conditions. A mixed ANOVA Type II for unbalanced data was performed to analyze whether the stimuli condition (HH, AA, HA, AH) and the diagnosis of the child and their interactions are associated with the frequency rate of happiness. Abbreviations: Dfn = degrees of freedom numerator; Dfd = degrees of freedom denominator; SSn = Sum of square numerator; SSd = Sum of square denominator; ges = generalized eta squared; Diagnosis = diagnosis of the child (TD or ASD); Condition = stimuli conditions (HH, AA, HA, AH); Dg:C = Interaction between diagnosis of the child and stimuli condition. GGe = Greenhouse-Geisser epsilon; HFe = Huynh-Feldt epsilon.
(PDF)

S2 Table Mixed ANOVA of reaction time response for each stimulus condition in both groups of parents. A mixed ANOVA Type II for unbalanced data was estimated to analyze whether the stimuli condition (HH, AA, HA, AH) or the diagnosis of the child (Group of parents) and their interactions are associated with the reaction time response. Abbreviations: Dfn = degrees of freedom numerator; Dfd = degrees of freedom denominator; SSn = Sum of square numerator; SSd = Sum of square denominator; ges = generalized eta squared; RT = Reaction time; Gr = Groups of parents (pTD or pASD); Cond = stimuli conditions (HH, AA, HA, AH).
(PDF)
