# Supplementary figures and images for: Differences in cortical processing of facial emotions in broader autism phenotype
Source: PLoS One. 2022 Jan 18;17(1):e0262004. doi: 10.1371/journal.pone.0262004 (PMC8765621; doi:10.1371/journal.pone.0262004)

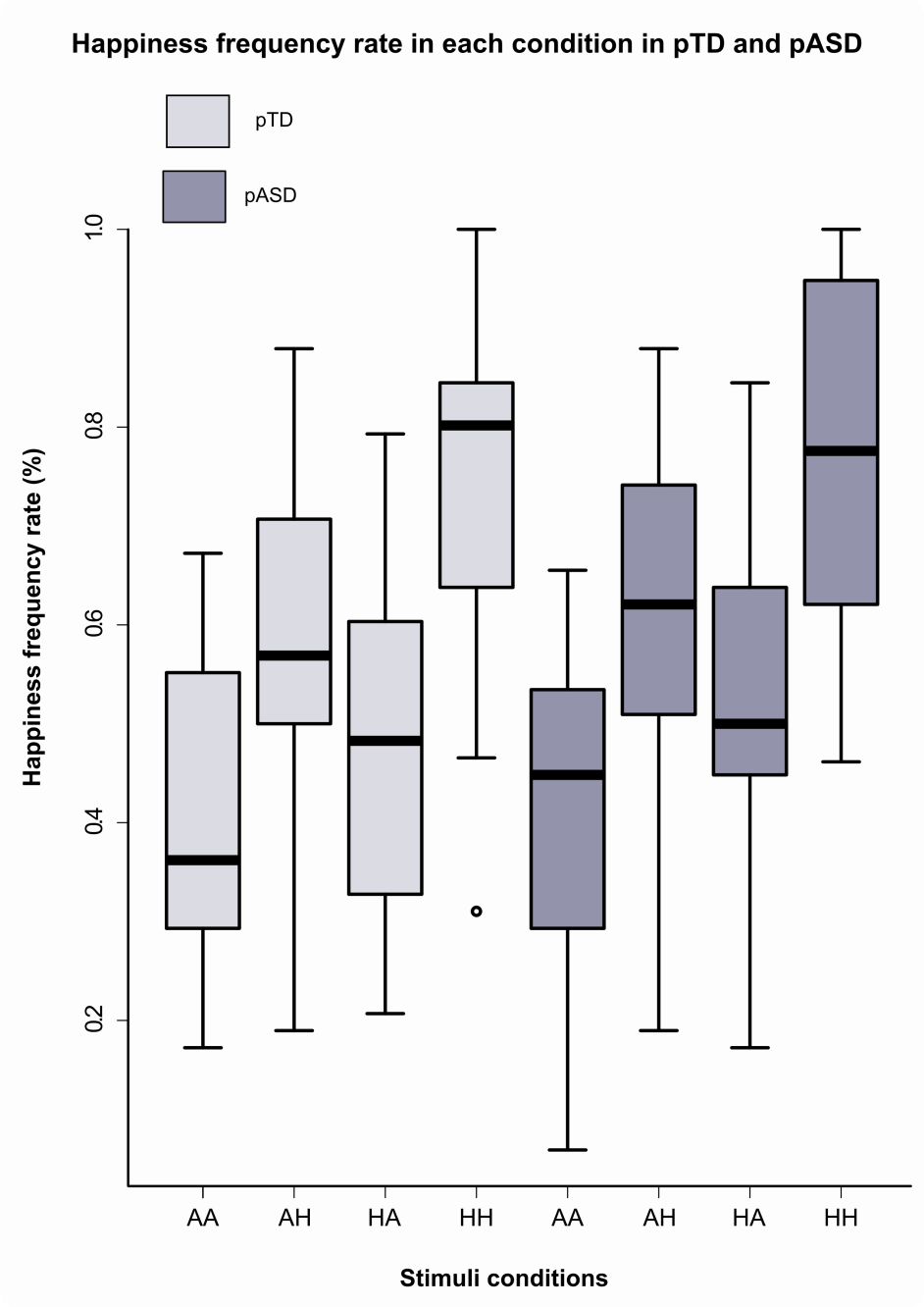

Supplement: S1 Fig — Four different stimuli were named as it follows: "AA", congruent stimulus for anger in which is displayed in both spatial frequencies; "HH", congruent stimulus for happiness; "AH", an incongruent stimulus where anger is presented in LSF and happiness in HSF; and finally, "HA", an incongruent stimulus where happiness is presented in LSF and anger in HSF. (TIF) [file pone.0262004.s001.tif]

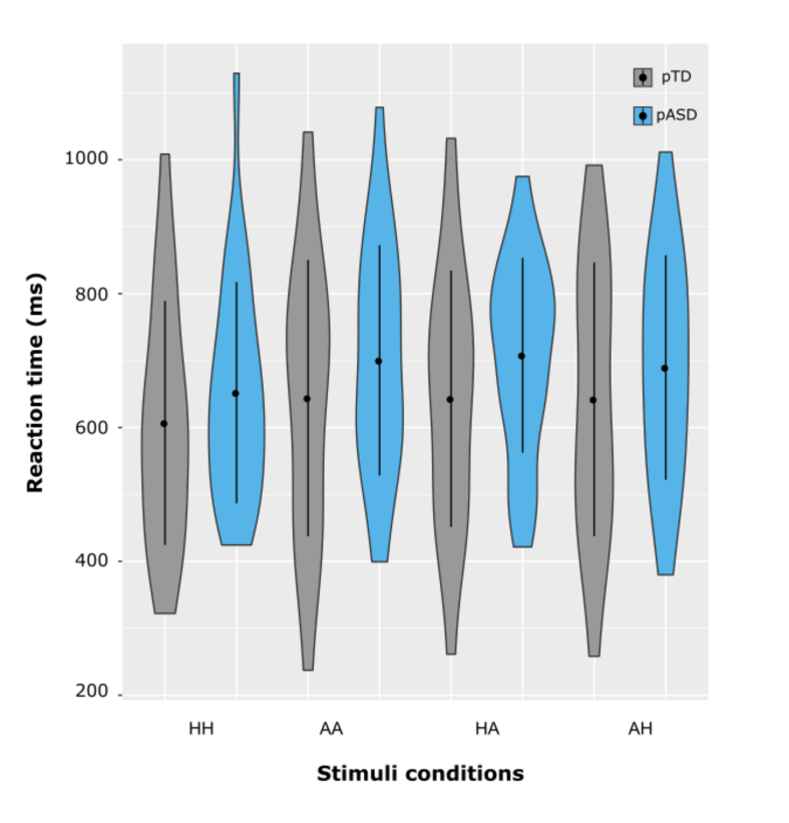

Supplement: S2 Fig — Reaction time for the subject’s choice of emotional stimuli in all stimuli conditions in pTD and pASD. Black point shows the mean of the distribution. Bars indicate standard deviation. (TIF) [file pone.0262004.s002.tif]
