# Supplementary material for: Differences in cortical processing of facial emotions in broader autism phenotype
Source: PLoS One. 2022 Jan 18;17(1):e0262004. doi: 10.1371/journal.pone.0262004 (PMC8765621; doi:10.1371/journal.pone.0262004)
Supplement: S1 Table — A mixed ANOVA Type II for unbalanced data was performed to analyze whether the stimuli condition (HH, AA, HA, AH) and the diagnosis of the child and their interactions are associated with the frequency rate of happiness. Abbreviations: Dfn = degrees of freedom numerator; Dfd = degrees of freedom denominator; SSn = Sum of square numerator; SSd = Sum of square denominator; ges = generalized eta squared; Diagnosis = diagnosis of the child (TD or ASD); Condition = stimuli conditions (HH, AA, HA, AH); Dg:C = Interaction between diagnosis of the child and stimuli condition. GGe = Greenhouse-Geisser epsilon; HFe = Huynh-Feldt epsilon. (PDF) [file pone.0262004.s003.pdf]

Mixed ANOVA frequency rate of happiness							
**Effect**	**Dfn**	**Dfd**	**SSn**	**SSd**	**F value**	**p value (< 0.05)**	**ges**
**Intercept**	1	41	55.272	0.827	2740	3.556e-39*	0.916
**Diagnosis**	1	41	0.032	0.827	1.594	0.213	0.006
**Condition**	3	123	2.879	4.234	27.8	7.969e-14*	0.362
**Dg : C**	3	123	0.007	4.234	0.07	0.975	0.001

Mauchly's Test for Sphericity		
**Effect**	**W**	**p value (< 0.05)**
**Condition**	0.3735657	2.28584e-07*
**Dg : C**	0.3735657	2.28584e-07*

Sphericity Corrections				
**Effect**	**GGe**	**p[GG] < 0.5**	**HFe**	**p [HF] (< 0.05)**
**Condition**	0.68225	3.811e-10*	0.718	1.445*
**Dg : C**	0. 68225	0.93	0.718	9.418
