# Supplementary material for: Differences in cortical processing of facial emotions in broader autism phenotype
Source: PLoS One. 2022 Jan 18;17(1):e0262004. doi: 10.1371/journal.pone.0262004 (PMC8765621; doi:10.1371/journal.pone.0262004)
Supplement: S2 Table — A mixed ANOVA Type II for unbalanced data was estimated to analyze whether the stimuli condition (HH, AA, HA, AH) or the diagnosis of the child (Group of parents) and their interactions are associated with the reaction time response. Abbreviations: Dfn = degrees of freedom numerator; Dfd = degrees of freedom denominator; SSn = Sum of square numerator; SSd = Sum of square denominator; ges = generalized eta squared; RT = Reaction time; Gr = Groups of parents (pTD or pASD); Cond = stimuli conditions (HH, AA, HA, AH). (PDF) [file pone.0262004.s004.pdf]

Mixed ANOVA Reaction time							
**Effect**	**Dfn**	**Dfd**	**SSn**	**SSd**	**F value**	**p value (< 0.05)**	**ges**
**Intercept**	1	41	5827	4879	489.6	2.05e-24*	0.918
**RT:Gr**	1	41	1196	4879	1.00	0.32	0.022
**RT:Cond**	3	123	6109	2937	8.52	3.43e-05*	0.011
**RT:Gr:RT:Cond**	3	123	2534.6	2937	0.35	7.86	0.000

Mauchly's Test for Sphericity		
**Effect**	**W**	**p value (< 0.05)**
**RT:Cond**	0.606	0.001*
**RT:Gr:RT:Cond**	0.606	0.001*

Sphericity Corrections				
**Effect**	**GGe**	**p[GG] < 0.5**	**HFe**	**p [HF] (< 0.05)**
**RT:Cond**	0.738	0.0002*	0.782	0.0001*
**RT:Gr:RT:Cond**	0.738	0.724	0.782	0.736
